# Further evidence for the existence of major susceptibility of nasopharyngeal carcinoma in the region near HLA-A locus in Southern Chinese

**DOI:** 10.1186/1479-5876-10-57

**Published:** 2012-03-22

**Authors:** Manli Zhao, Hongbing Cai, Xin Li, Hang Zheng, Xuexi Yang, Weiyi Fang, Longcheng Zhang, Ganguan Wei, Ming Li, Kaitai Yao, Xin Li

**Affiliations:** 1 Cancer Research Institute of Southern Medical University, Guangzhou 510515, China; 2 School of Chinese Traditional Medicine of Southern medical university, Guangzhou 510515, China; 3 Laboratory Medicine Center, Nanfang hospital of Southern Medical University, Guangzhou 510515, China; 4 Department of Oncology, Nanfang Hospital of Southern Medical University, Guangzhou 510515, China; 5 School of Biotechnology of Southern Medical University, Guangzhou 510515, China; 6 Department of Otolaryngology-Head and Neck Surgery, 303 Hospital of People's Liberation Army of China, Nanning 530021, China

**Keywords:** Nasopharyngeal carcinoma, Single nucleotide polymorphism, Human leukocyte antigen (HLA)

## Abstract

**Background:**

Nasopharyngeal carcinoma (NPC) is a multi-factorial malignancy closely associated with environmental factors, genetic factors and Epstein-Barr virus infection. Human leukocyte antigen (HLA) complex, specially the region near HLA-A locus, was regarded as a major candidate region bearing NPC genetic susceptibility loci in many previous studies including two recent genome-wide association (GWA) studies. To provide further evidence for the NPC susceptibility in the region near HLA-A locus based on other previous studies, we carried out a two-stage hospital-based case control association study including 535 sporadic NPC patients and 525 cancer-free control subjects from Guangdong, a high prevalence area of NPC in China.

**Methods:**

38 tag SNPs were initially selected by Heploview from the segment around HLA-A locus (from D6S211 to D6S510) and genotyped on GenomeLab SNPstream platform in 206 cases and 180 controls in the stage 1. Subsequently, the stage 1 significant SNPs and 17 additional SNPs were examined on another platform (Sequenom iPlex Assay) in another independent set of study population including 329 cases and 345 controls.

**Results:**

Totally eight SNPs from the segment from D6S211 to D6S510 within HLA complex were found to be significantly associated with NPC. Two of the most significant SNPs (rs9260734 and rs2517716) located near to HLA-A and HCG9 respectively were in strong LD with some other SNPs of this region reported by two previous GWA studies. Meanwhile, Meanwhile, novel independent susceptibility loci (rs9404952, Pcombined = 6.6 × 10^-5^, OR combined = 1.45) was found to be close to HLA-G.

**Conclusion:**

Therefore, our present study supports that the segment from D6S211 to D6S510 in HLA complex region might contain NPC susceptibility loci which indeed needs to be fully investigated in the future.

## Background

Nasopharyngeal carcinoma(NPC)is a sequamous cell carcinoma that originates from the epithelial lining of the nasopharynx. The incidence of NPC is low in most populations around the world but common in the southern part of China and other Southeast Asia region, where the incidence can reach 20 to 50 per 100,000 individuals[1,2], suggesting a strong association between specific genetic factors and NPC. Since 1974, many studies have indicated that some specific human leukocyte antigen (HLA) haplotypes and genes within the HLA complex are potentially associated with NPC [3-5]. Our previous meta-analysis [3] showed that populations in geographical areas at higher risk of developing NPC display HLA distribution patterns different and opposite from areas of low incidence. HLA complex is undoubtedly playing an important role in NPC predisposition, either by playing a functional role in modulating an innate and adaptive immune response against EBV, or as a marker of an unrelated predisposition locus in close linkage. Using high-resolution microsatellite mapping, Lu C.C. et al. [6] verified his previous study [7] and hypothesized the existence of a NPC susceptibility locus within a 132 Kb segment (from D6S211 to D6S510) containing the HLA-A locus in Taiwanese. Recently two independent research groups [8,9] conducted GWA studies in Taiwanese and southern Chinese respectively and consistently strengthened this hypothesis. Therefore, to further tested the hypothesis and provide further evidence for the possible existence of major susceptibility of Nasopharyngeal carcinoma in the region near HLA-A locus, we carried out a two-stage case-control association study to focus on the investigation of 132 Kb segment of interest with moderate sample size (535 cases and 525 control) in a southern Chinese population.

## Materials and methods

### Study subjects

This study was approved in advance by the institutional review Board of Southern Medical University. A total of 535 NPC patients with pathology-based diagnoses and 525 healthy unrelated controls without family or personal history of cancers and other major illnesses such as inflammation, diabetes, SLE, rheumatoid arthritis (RA), etc, were recruited from Jiangmen Center Hospital and Nanfang Hospital, Guangdong Province, between January 2005 and July 2008. The control individuals were frequency matched to the NPC cases by age, gender, geographic region, and ethnicity. Both cases and controls are of Cantonese origin from Southern China. All participants were informed by the aim of the study before participating in the study and required to sign the written informed consents. Peripheral blood samples were collected from all the participants and genomic DNA was extracted from peripheral blood samples using the Tiangen™ Genomic DNA Kit (Tiangen, China) following to the manufacturer's instructions and stored at -80°C before test.

### SNP selection, genotyping and quality control

The 132 kb segment between D6S211 and D6S510 (region from 29903 kb to 30050 kb) is our target region, from which 100 tag SNPs were selected based on Han Chinese (CHB) and Japanese (JPT) database in the HapMap Project [10] by using the Tagger program [11] to capture SNPs with a minimum minor allele frequency (MAF) of 0.05 and an *r*^2 ^of 0.80 or greater.

Of 100 tag SNPs picked out, 38 SNPs with C/T allele, which may be available for facilitating further DNA methylation analysis in our another project, were designed and genotyped for 206 cases and 180 controls in the first stage, using GenomeLab SNPstream genotyping platform (Beckman Coulter, Fullerton, California, USA) as previously described[12]. Subsequently, significant SNPs in the first stage, were designed and genotyped again in the second stage in another set of population composed of 329 cases and 345 controls, using the Sequenom iPLEX MassARRAY platform (Sequenom) as previously described[13]. To further increase the density of genetic markers, 17 additional SNPs were selected from those 100 tag SNPs for genotyping as well in the second stage. In the first stage, a sample call rate of over 95% was observed with 100% matching for quality control samples and blind replicates. In the second stage, the DNA sample quality control (QC) threshold was set at 90%.

### Statistical analysis

Deviations from the Hardy-Weinberg equilibrium (HWE) were firstly assessed using the Fisher's exact test or the Chi-square test. SNPs with HWE test p value > 10^-3 ^could be underwent further calculation. Association analysis based on unconditional logistic regression was carried out by figuring the odds ratio (OR) and 95% confidence interval (95% CI) for tag SNPs. The significance level was set at 0.05. All calculations were done with SPSS 13.0 software (SPSS, Chicago, IL).

The linkage disequilibrium (LD) and haplotype block analysis between significant SNPs in this study and other published SNPs located in HLA region (Additional file 1: Table S1) were calculated by Haploview software 4.2 using Han Chinese (CHB) and Japanese(JPT) data presented in the HapMap Project http://www.hapmap.org. D' and r^2 ^were used to describe LD relationship. The confidence interval method was adopted to set the haplotype block. This algorithm was taken from Gabriel et al. [14], in which 95% confidence bounds on D' were generated and each comparison was labeled "strong LD" (linkage disequilibrium), "inconclusive," or "strong recombination".

## Results

Demographic characteristics of both NPC cases and cancer-free controls were presented in Additional file 2: Table S2. Briefly, there was no significant difference in the distribution of age (P = 0.904) and gender (P = 0.874) between the cases and the controls. Among 535 NPC cases, 470 were Poorly differentiated SCC ( Squamous cell carcinoma), 58 Undifferentiated cancer, 7 Differentiated SCC. Both cases and controls are of Cantonese origin from Southern China.

A total of 38 tag SNPs included in the first stage were successfully genotyped. However, of them, five (rs1611650, rs9295822, rs9260759, rs1611442 and rs6457116) were found to have minor allele frequencies (MAFs) of less than 5%, and other two SNPs (Rs3202637 and rs2517681) were found to deviate from HWE. Therefore, the remaining 31 SNPs were further analyzed for genotype and allele frequencies.

As shown in Table 1 there were five SNPs (rs2517716, rs3869062, rs9260734, rs9260475 and rs9404952) showed significant differences in genotype and allele frequency adjusted for age and gender between NPC case and control group in the first stage. Although rs9260475 was unfortunately excluded as it was not in HWE, and rs3869062 showed a lower call rate (less than 90%) in the second stage, rs2517716, rs9260734 and rs9404952 still remained significant. Combination of these variant genotypes of stage 1 and stage 2 clearly showed that rs2517716 (OR _combined _= 1.62, P _combined _= 5.9 × 10-7), rs9260734 (OR _combined _= 1.67, P _combined _= 3.3 × 10-7), and rs9404952 (OR _combined _= 1.45, P _combined _= 6.6 × 10-5) were associated with a significantly increased risk of NPC.

**Table 1:** Significant SNPs identified in two independent stages of case-control studies

SNP	Position	Nearest	Alleles^a^	Stage I^e^			Stage II^f^			Combined^g^		
				
		Gene		MAF(%)^b^	P^c^	OR(95%CI)^d^	MAF(%)^b^	P^c^	OR(95%CI)^d^	MAF(%)^b^	P^c^	OR(95%CI)^d^
rs2517716	30025239	HLA-A	A/G	35.43	3 × 10^-3^	1.772(1.214-2.586)	39.6	4 × 10^-3^	1.420(1.120-1.800)	38.3	5.9 × 10^-7^	1.62(1.34-1.95)

rs9260734	30040645	HCG9	G/A	30.06	2 × 10^-3^	1.845(1.258-2.705)	32.8	4 × 10^-4^	1.535(1.209-1.95)	31.9	3.3 × 10^-7^	1.67(1.37-2.03)

rs9404952	29912144	HLA-G	A/G	34.86	1.7 × 10^-2^	1.528(1.080-2.163)	37.7	3 × 10^-3^	1.419(1.13-1.782)	36.7	6.6 × 10^-5^	1.45(1.21-1.75)

rs3869062^-^	30042870	HCG9	A/G	27.71	1.8 × 10^-2^	1.58(1.082-2.308)						

rs9260475^※^	30028105	HLA-A	T/C	37.57	1 × 10^-4^	2.034(1.426-2.902)						

^a ^Major/minor alleles as determined by allele frequency among genotyped controls; ^b ^Minor allele frequency among genotyped controls; ^c, d ^OR, odds ratio for major allele, adjusted by logistic regression for gender and age; ^e ^Stage 1, n = 206 cases and 180 controls; ^f ^stage 2, n = 329 cases and 345 controls; ^g ^combined stage 1 and 2, n = 535 cases and 525 controls. In stage 2, variations with ^- ^call rate < 90% and ^※ ^HWE < 0.001 were omitted.

**Figure 1:**
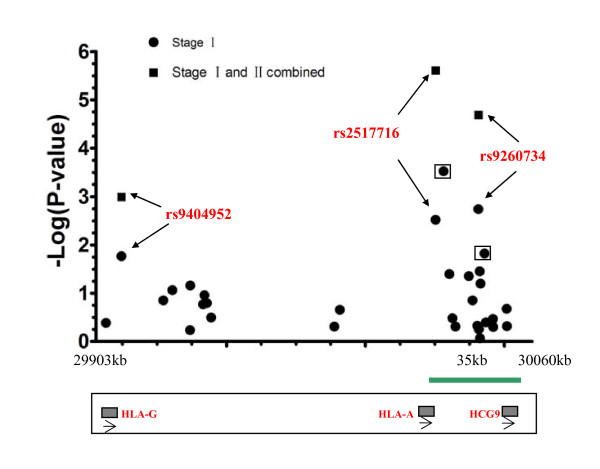
**Genotype association mapping in the region between D6S211 and D6S510**. The X-axis represents the locus of each SNP and the Y-axis represents the corresponding -Log10 (P-value) for each SNP locus. Two significant SNPs in black frame were omitted in the second stage.

Interestedly, rs9260734 has been previously reported to be associated with NPC risk in two GWA studies [8,9], but it was using less tag SNPs in a relatively small sample population that we could still detect its association effect. Moreover, rs9260734 and rs2517716 were found to map to a 35 kb region containing HCG9 and HLA-A (Table 1 Figure 1). For further confirming our result, we picked out 17 additional SNPs from this region for subsequent analysis. Of them, five (rs1632882, rs2571400, rs2735085, rs1632902 and rs16896742) displayed statistical significance (Additional file 3: Table S3) as well, strongly indicating the possible existence of candidate NPC susceptibility in this region.

**Figure 2:**
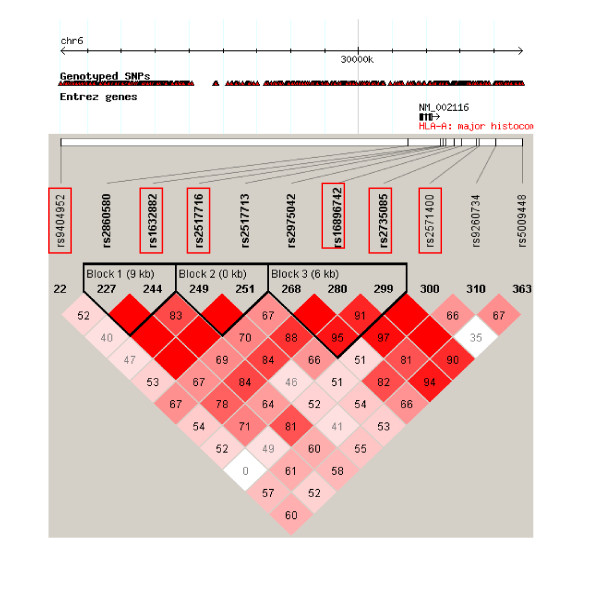
**LD between significant SNPs from our case-control study and other Significant SNPs from two NPC GWA studies based on CHB and JPT HapMap data**. LD was measured as D'. The red squares represent the LD between the two loci; the redder the color, the greater the linkage disequilibrium. Red cell without a number reflects D' = 1. Loci significant in the present study were marked in red frame in three blocks which were marked in black frame.

Furthermore, the linkage disequilibrium (LD) analysis was performed according to HapMap CHB and JPT data to measure LD for significant SNPs in our data set vs. significant SNPs in HLA complex from two recent GWA studies (Additional file 1: Table S1). Three LD blocks were formed separately (Figure 2). Some of significant SNPs were assigned into separate LD blocks together with some significant SNPs reported by Taiwan GWAS (rs2517713 and rs2975042) and the southern Chinese GWAS (rs2860580). Rs2517716 was in high LD with rs2517713 in block 2(D' = 1, r^2 ^= 0.7). Rs1632882, rs16896742 and rs2735085 of 17 additional SNPs and two GWAS rs2860580 and rs2975042 formed block 1 and 3. The LD coefficient D' was 1 and r^2 ^was 0.631 between rs2860580 and rs1632882. The LD coefficients (D') ranged from 0.952 to 1 and r^2 ^value ranged from 0.223 to 0.56 between rs2975042, rs16896742 and rs2735085. Therefore, although some of our significant loci were inconsistent with previous GWA studies, our LD analysis displayed strong LD relationships between them.

Rs9404952 as a novel SNP was located at another region downstream from HLA-G which has never been reported to be associated with NPC before. Accordingly, Rs9404952 did not merge with any blocks and the D' and r^2 ^between it and other SNPs were very low (D' from 0.006 to 0.676, r^2 ^from 0 to 0.271), suggesting that it might be an independent susceptibility factor.

## Discussion

Applying a two stage case-control analysis in southern Chinese, we demonstrated that multiple loci within the 132 Kb segment (from D6S211 to D6S510) were associated with NPC susceptibility in consistence with previous findings [6-9,15]; rs2517716 near HLA-A and rs9260734 near HCG9 were found significant, and a novel significant SNP, rs9404952, was close to the HLA-G gene suggesting HLA-G might be a new susceptible locus for NPC, and needs to be further researched.

The HLA-A gene is a HLA class I gene, which is involved in the presentation of foreign peptide antigens including EBV-derived peptides. HCG9 belongs to a multigene family (HCG) and is believed to be non-coding, but its function has not been determined. HLA-A and HCG9 were reportedly associated with the development of EBV-associated Hodgkin lymphoma [16] and infectious mononucleosis (IM) after primary EBV infection [17]. The association of HLA-A and HCG9 polymorphisms with NPC was reported in numerous studies including two GWA studies [6-9]. In addition, a recent association study was carried out by Li Y [15] validating Taiwan's GWAS and presenting three NPC susceptible SNPs, of which two located at HLA-A and HCG9 loci. In the current study, we found several NPC-associated risk SNPs near HLA-A and HCG9 locus as well. Interestedly, rs2517716 as one of the most significant SNPs was closed to the HLA-A locus and genetically linked to rs2517713 reported significant in the Taiwan group(r^2 ^= 0.7 and D = 1)based on Han Chinese (CHB) and Japanese (JPT) ancestries from HapMap data. Rs9260734 near HCG9 had been reported by both GWA studies in Taiwanese and Southern Chinese respectively [8,9], and also confirmed in the present study though it seems that its association signal might originate from the effects of HLA-A according to a previous research [9].

Moreover, in addition of the HLA-A and HCG9, our data for the first time showed that HLA-G might be involved in NPC susceptibility in southern Chinese. HLA-G belongs to the HLA non-classical class I heavy chain paralogues and seems to play a crucial role in immuno-tolerance mechanisms [18]. More recently, some studies have suggested that HLA-G plays an important role through the immune system in tumor development and progression [19-23]. It possesses the ability to suppress immune cell functions, such as NK- and CTL-mediated cytolysis and T-cell proliferative progress. HLA-G expression has been identified in several tumors such as breast cancer, glioblastoma, classical Hodgkin's lymphoma, and renal and lung cancers [19-23]. Ghandri N et al. [24] examined HLA-G gene polymorphisms in NPC for the first time using the restriction fragment length polymorphism-polymerase chain reaction (RFLP-PCR) and the amplification refractory mutation system-polymerase chain reaction (ARMS-PCR) method in Tunisian and found three nonsynonymous SNPs in codons not to associate with NPC susceptibility but implicate in NPC progression and survival. In the present study, rs9404952 located downstream of HLA-G gene showed a significant association with NPC though a less significant difference was found in the stage 1 (P = 0.017) possibly due to its small sample size. Hence, HLA-G might be a previously unreported candidate susceptibility locus for NPC among southern Chinese.

It is noted that previous GWA studies could not find the association of rs9404952 with NPC after scanning the whole human genome. The possible reasons are inferred as follow. First, due to its relatively larger P value (e.g., P > 10^-5^) in the preliminary stage of GWA studies, this SNP might be omitted from bioinformatics analysis. Second, such deviation might be resulted from genetic heterogeneity between populations. Furthermore, perhaps this SNP was not successfully genotyped by the genome-wide SNP-typing arrays due to some unclear reasons.

## Conclusion

In this study, we utilized tag SNPs according to Hapmap database to target the 132 kb segment between D6S211 and D6S510, and identified several loci significantly associated with NPC. In contrast to the GWA studies that examined large sample sets and more than thousands of genetic markers, our case-control analysis employed less tag SNPs in relatively small samples. Interestedly, we could also detect significant polymorphisms in HLA-A and HCG9, and even identified a new candidate susceptibility locus near HLA-G. Our study supported that the some HLA loci indeed have strong genetic effect for NPC.

Therefore, further investigations should be done in the future to expand the search for NPC susceptibility loci within or close to the HLA region, to explore functional loci depended on more advanced means, such as targeted exomic sequencing, and to further investigate the association between HLA-G locus and NPC risk.

## Competing interests

The authors declare that they have no competing interests.

## Authors' contributions

XL, KY and ML designed the study and modified manuscript as well as participated in data analysis and interpretation. MZ, XL and HC carried out most experimental studies and drafted the manuscript. WF and XY were in charge of the data assembly and statistic analysis. HZ collected blood sample materials and clinical data. GZ and GW mainly established and maintained sample database including clinical data. All authors read and approved the final manuscript.

## Supplementary Material

Additional file 1 Table 1**Significant loci within HLA-A region identified by previous GWAS**. Click here for file

Additional file 2 **Table 2 Distributions of select characteristics among patients and controls**. SCC: Squamous cell carcinoma. * Two-sided χ ^2 ^test. Click here for file

Additional file 3 **Table 3 Additional significant SNPs identified through the second stage**. ^a ^Major/minor alleles as determined by allele frequency among genotyped controls; ^b ^Minor allele frequency among genotyped controls; ^c, d ^OR, odds ratio for major allele, adjusted by logistic regression for gender and age. Click here for file
