# Supplementary material for: Further evidence for the existence of major susceptibility of nasopharyngeal carcinoma in the region near HLA-A locus in Southern Chinese
Source: J Transl Med. 2012 Mar 22;10:57. doi: 10.1186/1479-5876-10-57 (PMC3383544; doi:10.1186/1479-5876-10-57)
Supplement: Additional file 1 — Table 1Significant loci within HLA-A region identified by previous GWAS. [file 1479-5876-10-57-S1.DOC]

**Supplementary table 1**.

| SNP | Risk allele | OR(95% CI) | P-value | Position | Nearest Gene | GWA Study Reference |
| --- | --- | --- | --- | --- | --- | --- |
| rs2517713 | A | 1.88 (1.65–2.15) | 3.9E-20 | 30026078 | HLA-A | Tse, K.P., et al. |
| rs2975042 | A | 1.86 (1.63–2.13) | 2.54E-08 | 30028515 | HLA-A | Tse, K.P., et al. |
| rs9260734 | G | 1.85 (1.61–2.12) | 2.49E-07 | 30040645 | HCG9 | Tse, K.P., et al. |
| rs3869062 | A | 1.78 (1.55–2.05) | 1.58E-06 | 30042870 | HCG9 | Tse, K.P., et al. |
| rs5009448 | G | 1.72 (1.51–1.96) | 9.41E-06 | 30048467 | HCG9 | Tse, K.P., et al. |
| rs2860580 | C | 1.72（1.61-1.82） | 4.88E-67 | 30014670 | HLA-A | Bei, J., et al |
