# Supplementary material for: Further evidence for the existence of major susceptibility of nasopharyngeal carcinoma in the region near HLA-A locus in Southern Chinese
Source: J Transl Med. 2012 Mar 22;10:57. doi: 10.1186/1479-5876-10-57 (PMC3383544; doi:10.1186/1479-5876-10-57)
Supplement: Additional file 2 — Table 2 Distributions of select characteristics among patients and controls. SCC: Squamous cell carcinoma. * Two-sided χ 2 test. [file 1479-5876-10-57-S2.DOC]

**Supplementary Table 2.**

| Characteristics | Stage 1 | |  |  | Stage 2 | | |  | | |  | Combined | | | | | | |
| --- | --- | --- | --- | --- | --- | --- | --- | --- | --- | --- | --- | --- | --- | --- | --- | --- | --- | --- |
|  | Cases  (n=206) | Controls  (n=180) | *P** |  | Cases  (n=329) | | Controls  (n=345) | *P** |  | | | | Cases  (n= 535) | | Controls  (n=525) | | *P** | |
|  | n (%) | n (%) |  |  | n (%) | | n (%) |  |  |  |  |  | n (%) | | n (%) | |  |  |
| Gender |  |  |  |  | |  |  |  | |  | | | |  | |  | |  |
| Male | 148(71.1) | 120(66.7) |  |  |  | 241(73.3) | 264(76.5) |  | |  |  |  |  | 389(72.7) | | 384(73.3) | |  |
| Female | 58(27.9) | 60(33.3) | 0.271 |  | | 88(26.7) | 81(23.5) | 0.328 | |  | | | | 146(27.3) | | 141(26.9) | | 0.874 |
| Age(years) |  |  |  |  |  |  |  |  | |  |  |  |  |  | |  | |  |
| ≤55 | 146(70.9) | 138(76.7) |  |  |  | 218(66.3) | 221(64.1) |  | |  |  |  |  | 364(68.0) | | 359(68.4) | |  |
| >55 | 60(29.1) | 42(23.3) | 0.198 |  |  | 111(33.7) | 124(35.9) | 0.548 | |  |  |  |  | 171(32.0) | | 166(31.6) | | 0.904 |
| Histological type |  |  |  |  |  |  |  |  | |  |  |  |  |  | |  | |  |
| Poorly differentiated SCC | 180(87.4) |  |  |  |  | 290(88.1) |  |  | |  |  |  |  | 470(87.9) | |  | |  |
| Undifferentiated cancer | 22(10.7) |  |  |  |  | 36(10.9) |  |  | |  |  |  |  | 58(10.8) | |  | |  |
| Differentiated SCC | 4(1.9) |  |  |  | | 3(0.9) |  |  | |  | | | | 7 (1.3) | |  | |  |
