# Supplementary material for: Further evidence for the existence of major susceptibility of nasopharyngeal carcinoma in the region near HLA-A locus in Southern Chinese
Source: J Transl Med. 2012 Mar 22;10:57. doi: 10.1186/1479-5876-10-57 (PMC3383544; doi:10.1186/1479-5876-10-57)
Supplement: Additional file 3 — Table 3 Additional significant SNPs identified through the second stage. a Major/minor alleles as determined by allele frequency among genotyped controls; b Minor allele frequency among genotyped controls; c, d OR, odds ratio for major allele, adjusted by logistic regression for gender and age. [file 1479-5876-10-57-S3.DOC]

**Supplementary table 3**

| SNP | Position | Nearest Gene | Alleles^a^ | MAF(%)^b^ | P^c^ | OR(95%CI)^d^ |
| --- | --- | --- | --- | --- | --- | --- |
| rs1632882 | 30024347 | HLA-A | C/A | 34.8 | 3×10^-3^ | 1.422(1.127-1.795) |
| rs2571400 | 30035701 | HCG9 | G/C | 47.7 | 4×10^-3^ | 1.369(1.104-1.697) |
| rs2735085 | 30035074 | HLA-W | C/T | 31.4 | 2×10^-3^ | 1.461(1.146-1.864) |
| rs1632902 | 30005391 | HCG4P6 | C/G | 16.5 | 2.5×10^-4^ | 2.027(1.389-2.950) |
| rs16896742 | 30030719 | HLA-A | A/G | 40.6 | 3×10^-3^ | 1.382(1.114-1.716) |
